# ICTV Virus Taxonomy Profile: *Sunviridae* 2023

**DOI:** 10.1099/jgv.0.001896

**Published:** 2023-10-13

**Authors:** Jens H. Kuhn, Gael Kurath, Yuri I. Wolf, Timothy H. Hyndman

**Affiliations:** ^1^​ Integrated Research Facility at Fort Detrick, National Institute of Allergy and Infectious Diseases, National Institutes of Health, Fort Detrick, Frederick MD 21702, USA; ^2^​ United States Geological Survey Western Fisheries Research Center, Seattle, WA 98115, USA; ^3^​ National Center for Biotechnology Information, National Library of Medicine, National Institutes of Health, Bethesda MD 20894, USA; ^4^​ School of Veterinary Medicine, Murdoch University, Murdoch, WA 6150, Australia

**Keywords:** ICTV Report, taxonomy, *Sunviridae*, sunshinevirus, Sunshine Coast virus

## Abstract

*Sunviridae* is a family of negative-sense RNA viruses with genomes of about 17.2 kb that have been found in snakes. The sunvirid genome comprises nonsegmented RNA with six open reading frames (ORFs) >1 kb that are predicted to encode six proteins. This is a summary of the International Committee on Taxonomy of Viruses (ICTV) Report on the family *Sunviridae*, which is available at ictv.global/report/sunviridae.

## Virion

Infectivity is lost following treatment with chloroform and so it is assumed that sunvirids are enveloped particles [1] (Table 1).

**Table 1.** Characteristics of members of the family *Sunviridae*

Example	Sunshine Coast virus (JN192445), species *Sunshinevirus reptilis*, genus *Sunshinevirus*
Virion	Enveloped, spherical
Genome	A nonsegmented negative-sense RNA of about 17.2 kb
Replication	Unknown
Translation	Unknown
Host range	Squamate reptiles (pythonid and boid snakes)
Taxonomy	Realm *Riboviria*, kingdom *Orthornavirae*, phylum *Negarnaviricota*, class *Monjiviricetes*, order *Mononegavirales*; the family includes the genus *Sunshinevirus* and the species *Sunshinevirus reptilis*

## Genome

**Fig. 1.**
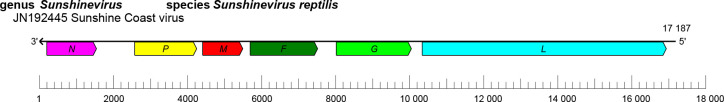
Genome length, organization and position of open reading frames (ORFs) of Sunshine Coast virus. ORFs are indicated as boxes, coloured according to the predicted protein function (*N*, nucleocapsid protein gene; *P*, phosphoprotein gene; *M*, matrix protein gene; *F*, fusion protein gene; *G*, glycoprotein gene; *L*, large protein gene).

The sunvirid genome is a nonsegmented negative-sense RNA with a total length of about 17.2 kb (Fig. 1). The genome contains six ORFs that are >1 kb, which encode six proteins: nucleocapsid protein (N), phosphoprotein (P), matrix protein (M), fusion protein (F), glycoprotein (G) and a large (L) protein containing an RNA-directed RNA polymerase domain (Fig. 1). Each ORF is flanked by transcriptional start and stop sequences with similar motifs to those of paramyxovirids [1, 2].

## Replication

Unknown.

## Pathogenicity

Sunshine Coast virus is associated with neurorespiratory disease in snakes [3]. Pathological changes associated with infection are most often detected in brain tissue and typically consist of white matter spongiosis of the hindbrain in the absence of gross lesions. Virus can be detected in the mouths and cloacas of infected snakes, suggesting virus transmission via oral and/or cloacal secretions. Snake embryos and allantois and amnion membranes may contain viral RNA but RNA-positive embryos are not known to be viable, suggesting inefficient or self-limiting vertical transmission [3, 4].

## Taxonomy

**Fig. 2.**
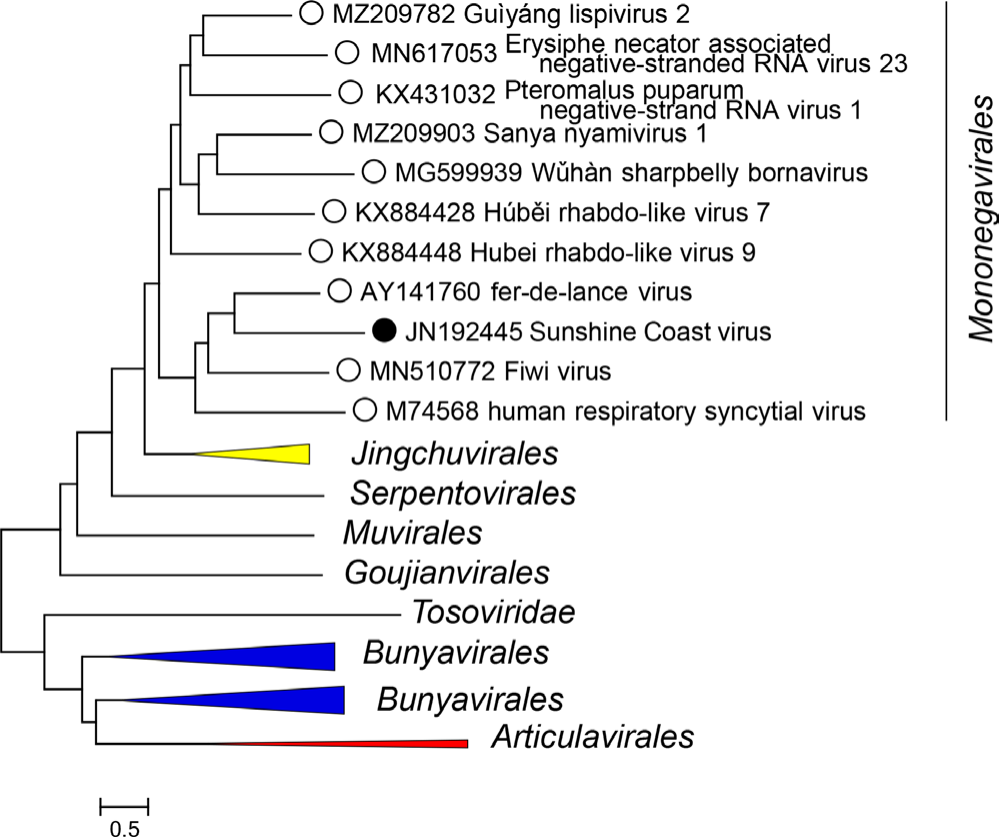
Phylogenetic relationships of Sunshine Coast virus. A phylogenetic tree was reconstructed for an alignment of the RNA-directed RNA polymerase core domains of selected members of the *Negarnaviricota* (one per family, see ICTV Report for details) using the FastTree program [5]. A black dot indicates Sunshine Coast virus.

Current taxonomy: ictv.global/taxonomy. The family *Sunviridae* includes the genus *Sunshinevirus* and the species *Sunshinevirus reptilis* for viruses that infect snakes. Sunshine Coast virus is most closely related to mononegaviral filovirids, paramyxovirids, and pneumovirids (Fig. 2).

## Resources

Full ICTV Report on the family *Sunviridae*: ictv.global/report/sunviridae.
